# The do’s, don’ts and don’t knows of redressing differential attainment related to race/ethnicity in medical schools

**DOI:** 10.1007/s40037-021-00696-3

**Published:** 2021-12-29

**Authors:** Molly Fyfe, Jo Horsburgh, Julia Blitz, Neville Chiavaroli, Sonia Kumar, Jennifer Cleland

**Affiliations:** 1 Medical Education Innovation and Research Centre, Imperial College London, London, UK; 2 Centre for Higher Education Research and Scholarship, Imperial College London, London, UK; 3 Centre for Health Professions Education, Faculty of Medicine and Health Sciences, Stellenbosch University, Stellenbosch, South Africa; 4 Australian Council for Educational Research, Melbourne, Australia; 5 Lee Kong Chian School of Medicine, Nanyang Technological University, Singapore, Singapore

**Keywords:** Differential attainment, Ethnicity, Racism, Medical education, Assessment, Learning environment

## Abstract

**Introduction:**

Systematic and structural inequities in power and privilege create differential attainment whereby differences in average levels of performance are observed between students from different socio-demographic groups. This paper reviews the international evidence on differential attainment related to ethnicity/race in medical school, drawing together the key messages from research to date to provide guidance for educators to operationalize and enact change and identify areas for further research.

**Methods:**

Authors first identified areas of conceptual importance within differential attainment (learning, assessment, and systems/institutional factors) which were then the focus of a targeted review of the literature on differential attainment related to ethnicity/race in medical education and, where available and relevant, literature from higher education more generally. Each author then conducted a review of the literature and proposed guidelines based on their experience and research literature. The guidelines were iteratively reviewed and refined between all authors until we reached consensus on the Do’s, Don’ts and Don’t Knows.

**Results:**

We present 13 guidelines with a summary of the research evidence for each. Guidelines address assessment practices (assessment design, assessment formats, use of assessments and post-hoc analysis) and educational systems and cultures (student experience, learning environment, faculty diversity and diversity practices).

**Conclusions:**

Differential attainment related to ethnicity/race is a complex, systemic problem reflective of unequal norms and practices within broader society and evident throughout assessment practices, the learning environment and student experiences at medical school. Currently, the strongest empirical evidence is around assessment processes themselves. There is emerging evidence of minoritized students facing discrimination and having different learning experiences in medical school, but more studies are needed. There is a pressing need for research on how to effectively redress systemic issues within our medical schools, particularly related to inequity in teaching and learning.

## Definitions of do’s, don’ts and don’t knows

### Do’s

Educational activity for which there is evidence of effectiveness

### Don’ts

Educational activity for which there is evidence of no effectiveness or of harm (negative effects)

### Don’t knows

Educational activity for which there is no evidence of effectiveness

## Introduction

In essentially all societies some groups hold more privileges or social resources (power, status, wealth, and opportunity) than others, leading to inequality between groups [1]. Privilege is then maintained and reproduced by norms, assumptions and systems of power. The systematic, or structural, inequity of power and privilege create differential attainment. Differential attainment refers to unexplained variation in average (not individual) levels of performance on educational assessments between students from different societal groups. These students also report different experiences of education (see later for further discussion).

While student socio-demographic characteristics are statistically correlated with differential outcomes, these should not be interpreted as causal. It is the experience of minoritization (defined later) that is likely causally associated with differential attainment, not the socio-demographic characteristics themselves [2–4]. Interpreting causality based on student demographic characteristics contributes to deficit thinking [2] in which students from lower performing groups are seen as in some way deficient due to their backgrounds, even when acknowledging the social and institutional drivers of minoritization for these identities.

Minoritization occurs in medicine, which privileges some people, cultures and practices above others [3–5]. As with education generally [6–8], those who are not from privileged groups are often disadvantaged in their educational experiences and outcomes. Differential attainment in medical education is evidenced across various intersecting characteristics such as ethnicity (and race) [9–14], age [11, 12], gender [11, 15, 16] and disability/neurodiversity [17, 18]. Differential attainment is observed across multiple types of outcomes, including clinical assessments [10, 14], timely course completion [11], clerkship grades [19], in course grade point average [13], narrative descriptions of student performance [20], multiple-choice exams [14] and honour society inductions [9, 12]. Differential attainment in medical education can hinder individuals’ learning experiences and career progression [14, 21, 22]; limit diversity of the health workforce [23–25]; and ultimately impact on patient care [26].

Synthesizing the literature relating to differential attainment in medical education across all minoritized groups was too broad for any one review, particularly given the structures which underpin inequality vary across contexts [27]. We chose instead to focus on differential attainment related to ethnicity/race because of the ongoing crisis of racism: we were preparing these guidelines during a time of turbulence and heightened awareness of systemic racism. The Covid-19 pandemic exacerbated health and other inequalities [28] and the Black Lives Matter movement had increased awareness of racial inequality and white privilege across the globe [29]. This paper was therefore written at a time in which our understanding and awareness of racial inequality was rapidly evolving resulting in increased responsiveness, refined conceptualizations and new terminology. Moreover, the dominant descriptions in the literature were associated with ethnicity/race. Note that race and ethnicity are not causally related to differential attainment, but rather reflect how privilege and discrimination manifest in educational settings and practices [3]. Moreover, race and ethnicity are socially constructed and contested constructs that tend to be defined by those in power in a particular context [27, 30]. The result is that racial and ethnic categories are imprecise and context dependent, shifting over time and between settings [27]. This is reflected in the literature on differential attainment: what groups are minoritized varies between studies and contexts.

### Aim

In many countries there is an ethical and legal imperative to address inequalities in medical education [31, 32]. To aid this endeavour, in this paper we review the international evidence on differential attainment related to ethnicity/race in medical school, drawing together the key messages from research to date to provide guidance for educators to operationalize and enact change and identify areas for further research. Specifically, we present evidence-based guidelines for practices in assessment and the learning environment to support equity of experience and attainment at medical school. We preface our interpretation of the literature with a synthesis of how differential attainment is situated in the literature, as a necessary foundation for providing guidelines for its redress.

## Methods

These guidelines are based on consensus of expert opinion across medical educators based in five countries, all of whom have published scholarship related to differential attainment [5, 33–38]. Authorship opinion identified areas of conceptual importance within differential attainment (learning, assessment, and systems/institutional factors) which were then the focus of a targeted review of the literature on differential attainment in medical education and, where available and relevant, literature from higher education more generally. We utilized an iterative process similar to that outlined in previously published papers for this Guidelines series [39, 40].

Our review of the literature was guided initially by the concept of fair assessment, as articulated by Lucey [41]: “Equity in assessment is present when all students have fair and impartial opportunities to learn, be evaluated, coached, graded, advanced, graduated, and selected for subsequent opportunities based on their demonstration of achievements that predict future success in the field of medicine, *and* that neither learning experiences nor assessments are negatively influenced by structural or interpersonal bias related to personal or social characteristics of learners or assessors” (p. 599). However, given differential attainment is created by structural inequalities we kept our search broad, to ensure consideration of learning and systems/institutional factors.

### Our focus

Our review focused on differential attainment related to ethnicity/race in medical school. Medical school is the first formal stage of medical training and unequal attainment that manifests at this stage underpins a growing chasm of disparities throughout the continuum of medical training and practice [21, 42]. Addressing differential attainment in medical school is thus critical to achieving equity throughout the continuum of medical training and practice.

Selection is positioned as the first assessment of medical school [43] and differential attainment is very relevant to being able to apply for or be accepted to medical school. However, issues related to the fairness of selection processes have been reviewed elsewhere [43]. Our focus in this paper was medical students (that is, those individuals who have been successful in obtaining a place at medical school), and the assessments and learning environment once in medical school.

### Terminology

Many different terms are used in the literature to categorize groups from different racial and ethnic backgrounds (e.g., BME [Black and Minority Ethnic], BIPOC [Black, Indigenous and People of Colour]). In this paper, we employ the more contemporary term “minoritized”, defined by Selvarajah et al. [27] as “individuals and populations, including numerical majorities, whose collective cultural, economic, political and social power has been eroded through the targeting of identity in active processes that sustain structures of hegemony.” (p. 3). This term recognizes power imbalance and acknowledges systemic acts of discrimination, exclusion and omission across multiple settings. Differential attainment and minoritization are not the same: rather differential attainment is one of a number of potential consequences of minoritization and can contribute to further minoritization.

A variety of institutional and social factors have been suggested to underpin minoritization and differential attainment in higher education: structural inequalities related to power and race [44, 45], discrimination and microaggressions [44, 46], assessment practices [14, 47], relationships and access to social capital (resources and opportunities) [48, 49] and the nature of the learning environment [44, 50, 51]. Although we present our findings in 13 individual “Do, Don’t and Don’t Know” categories, it is important to note that, for students from minoritized backgrounds, these categories and the structural and systemic factors underpinning differential attainment intersect and overlap [52].

The terms differential awarding instead of differential attainment, and awarding gap rather than attainment gap, have been proposed recently as a way to shift the focus more toward institutional responsibility [21, 53]. However, for the purposes of this paper we kept the arguably more controversial term of differential attainment. Our reasons for doing so were twofold. First, this is the term used widely in the literature reviewed. Second, the message of shifting focus comes out clearly from the Guidelines.

### Process

Following agreement upon the topic and the definitions of fairness in assessment and minoritization provided above [41], each author conducted a review of the literature relevant to the topic and definitions discussed above, then used that review to inform a proposal for guidelines. While this is not a systematic review, for transparency we provide an overview of how we identified relevant literature. Searches were conducted in PubMed and Google Scholar, using the terms “assessment”, “medical” in combination with “bias”, “culturally responsive assessment”, “differential attainment”, “disparities”, “diversity”, “ethnicity”, “ethnic minorities” and “fairness”, and combinations thereof. We set no start date to the search. Searches were conducted iteratively between June 2019 and June 2021. Authors also drew on their knowledge of the literature to ensure all relevant papers were included. While we focused on identifying empirical studies (qualitative and quantitative), we also drew on perspective pieces to help identify how differential attainment related to ethnicity/race has been conceptualized in the literature. Our own limitations meant we searched only for English-language papers. Many of the papers were quantitative, particularly those examining assessment practices and processes. However, the papers which focused on educational experiences were more diverse, reflecting quantitative, qualitative and mixed methods approaches. Overall, we drew on 124 papers to develop these guidelines.

MF then compiled an initial list of Do’s, Don’ts and Don’t Knows with input from the wider team. Each author continued to add to and refine this list. Through discussions in person, via email and by Zoom or Skype, the lists were reviewed and refined until we reached consensus on the Do’s, Don’ts and Don’t Knows. We took care to ensure that “Don’ts” were not just the opposite of “Do’s”, and vice-versa. When evidence was lacking or conflicting, an item was allocated to the “Don’t Know” category. This is a valuable category in terms of setting future research agendas.

The paper was developed as a group effort, with each author taking a number of items from the original list, exploring the wider education literature on each of these items, then producing a summary and critique of that literature. These narratives were drawn together in a draft document by MF and JH. All authors then contributed further comments and edits, and reached consensus through discussing the narrative and rating for each guideline (see Table 1). The evolving drafts of the document were edited extensively by JC.

**Table 1:** Criteria for strength of recommendation

Strong	A large and consistent body of evidence
Moderate	Solid empirical evidence from one or more papers plus consensus of the authors
Tentative	Limited empirical evidence, but clear consensus of the authors

### Positionality and reflexivity

This review is a qualitative synthesis of the literature and as such it was important for us to reflect on our backgrounds and social worlds [54], and how these may have shaped our interpretation of the literature. Our identities are nuanced and intersectional in relation to ethnicity, gender, learning experiences and disciplinary backgrounds, research interests and personal life courses [55–57]. We are now based in five countries, each of which have different medical education and training systems as well as representing very different contexts in terms of power and privilege, and how access and opportunity are distributed in society [1]. These differences led to lively discussions on the complex topic of differential attainment, as well as (sometimes painful) individual and collective critiques of our own consciousness of structural inequality and biases, convergences and differences [54].

## Results

We drew from the research to gain insight into how differential attainment manifests, how it is experienced by medical students and what strategies, or approaches, can be used to redress inequalities. We first describe how ‘attainment’ is situated in the literature, interrogate how race/ethnicity are conceptualized and operationalized in medical education research, and very briefly summarize the research describing the causes of differential attainment. We then present our guidelines (Do’s and Don’ts) based on evidence for strategies and approaches to redress differential attainment. Lastly, we present our ‘Don’t Knows’ as priority issues needing further development and research.

### Attainment

Attainment in education is a broad concept, including performance on educational assessments, achieving a degree, degree outcomes and continuation into postgraduate study and employment [47, 48].

Within medical education, practices of assessment and approaches to research into assessment comes from a variety of epistemic positions. Much of the research that quantifies assessment outcomes and differences in these (differential attainment) is rooted in positivist and post-positivist epistemologies. On the other hand, there is also prevalence of assessment practices that emphasize plurality in views, multiple perspectives, and the validity of subjectivity (e.g. [49, 50]). Within this paper we draw on research from a range of philosophical positions and these guidelines necessarily reflect the philosophical stances of the research on which they are based.

### Conceptualization of race and ethnicity in the research on differential attainment

As stated earlier, our focus is the relationship between attainment and ethnicity/race at medical school. The quantitative research on differential attainment in medical education focuses on how students minoritized on the basis of race/ethnicity perform compared with a reference group. Differential attainment between racially/ethnically minoritized and norm groups (see earlier) is reported across multiple types of outcomes, including clinical assessments [10, 14], timely course completion [11], clerkship grades [19], grade point average [13], narrative descriptions of student performance [20], multiple-choice exams [14] and honour society inductions [9, 12]. Although attrition from university programmes is often used as an indicator for differential attainment in university programmes [51], only a few of these studies have looked at course completion as an outcome measure because attrition from medical school is relatively low [10, 13].

Which identities are minoritized in studies varies according to the institutional and societal context. For example, one of the aforementioned studies, from New Zealand, investigated academic outcomes across “Maori”, “Pacific”, and “non-Maori non-Pacific” students [13] while one from the USA used a grouping of “Black or African American, Hispanic, American Indian/Alaska Native, or Native Hawaiian/Pacific Islander” [21]. However, while the groups compared are context-specific, the patterns are common. There is a consistent pattern of minoritized medical students having lower performance on assessments globally (e.g. [9–15, 20, 21, 36, 48, 51]).

In most studies student assignment into a group is via self-identified race and ethnicity, often through self-selection into pre-determined categories based on skin colour, and geographic or cultural identity (e.g., Black Caribbean). These self-identifications of race are then often amalgamated by researchers into heterogeneous categories, with categories such as “white” or “Western” often used as the norm reference group (e.g. [13–15, 37]). The reasons for this are ostensibly statistical: where there are many groups with small numbers, analysis is not possible without re-categorization. While pragmatic, this approach has been criticized for failing to recognize the diversity and intersectionality of student identities and experiences within such broad groupings [47, 50, 56, 57]. Moreover, decisions around assigning a “norm” and “minoritized” groups are not neutral, and likely reflect the positionality of the research team as well as the logistics of a dataset.

As we hope is clear in the introduction to this paper, inequalities and minoritization occur both inside higher education practices and within the wider societies in which medical schools are located. The complexity of institutional (e.g., assessment and teaching approaches) and societal practices (e.g., poverty and longitudinal educational opportunity), norms, privileges and biases that contribute to differential attainment make it challenging to understand the fundamental causes of differential attainment and what actions will effectively redress it. Nevertheless, based on analysis and collation of the literature, we bring our interpretation and understanding of pedagogical practices which seem to contribute and may need to be addressed in relation to differential attainment in minoritized medical students to the guidelines.

A summary of the 13 “Do’s, Don’ts and Don’t Know” guidelines is presented in Table 2. We have teased out how these guidelines individually have influence, but the bigger picture is derived from how they may inter-relate.

**Table 2:** Summary of guidelines, including strength of evidence and broad area of focus

	Recommendations	Strength	Area of focus
*Do’s*			
1	Ensure assessments are fair and defensible	Strong	Assessment
2	Use robust and recognized analytic approaches to scrutinize assessments and assessment data	Strong	Assessment
3	Sample broadly across assessment types and assessors to minimize bias	Strong	Assessment
4	Include the concept of ‘cultural validity’ in the design, development and review of assessments	Moderate	Assessment
5	Do recognize unequal privilege and power in the learning environment as causes of differential attainment and take steps to mitigate their negative impacts	Strong	Learning
6	Medical schools must take responsibility for their role in creating and perpetuating differential attainment	Strong	Systems/institutional level
7	Recruit and promote under-represented faculty	Strong	Systems/institutional level
*Don’ts*			
8	Don’t assume that minoritized students will have the same exposure to and familiarity with particular assessment methods as their non-minoritized peers	Tentative	Learning
9	Don’t implement ‘diversity’ practices which place unacknowledged and unrewarded burdens on minoritized faculty and students	Moderate	Systems/institutional level
10	Don’t attribute to the individual what is systematic	Strong	Systems/institutional level
*Don’t know’s*			
11	Are supportive interventions for specific groups effective in respect of assessment outcomes?	–	Assessment
12	To what extent do professionalism norms and assessments of professionalism discriminate against minoritized students?	–	Assessment
13	How can formal curricula influence differential attainment?	–	Learning

#### Do’s

##### Guideline 1.

*Ensure assessments are fair and defensible* **(Strong)**

Fairness is fundamental to good assessment yet remains elusive to define [58–60]. We define fair assessments as those which offer learners similar or equitable opportunity to demonstrate their knowledge, understanding and competence with minimal impact from external factors, such as gender, race or ethnicity. Such external factors might be mediated by the test question context or assumptions in the case of written exams, or by examiner bias (implicit or otherwise) in the case of performance exams. Importantly, fairness does not mean or imply that assessments need to pose a similar challenge to every student; differential knowledge and understanding is a legitimate discriminator [58, 59]. In fair assessments, however, it will be the only significant discriminator.

Proactively ensuring valid and defensible assessments which minimize the impact of extraneous (or “construct-irrelevant”) factors on student performance by paying attention to the basic elements of assessment will be helpful in respect of fairness. Such factors traditionally include design, question writing, peer review, test construction, appropriate scoring and post hoc analysis [59], as well as blueprinting, adequate sampling, careful planning and a programme-level focus [60]. But each of these aspects of assessment carries certain epistemological assumptions and constructed notions which should also be interrogated in determining how fair assessments are, in the same way that discourses of ‘merit’ involve far-reaching assumptions and potential biases [61]. We propose that examination content and underlying assumptions must be routinely analyzed and reviewed to ensure they do not present inappropriate progression barriers for any group.

##### Guideline 2.

*Use robust and recognized analytic approaches to scrutinize assessments and assessment data *(**Strong**)

Assessment data should be routinely analyzed to identify differential attainment. Item response theory (IRT) can provide information about items (e.g., exam questions) which function differently across subgroups [62–64]. One IRT approach, Differential Item Functioning (DIF), can be used to see if certain questions in a written or objective structured clinical examination (OSCE)-style examination explain group-level differences in performance [65, 66]. Where test questions discriminate between groups, these items can be revised or removed, and replaced with less biased questions. Recent studies have indicated that another IRT approach, Many Faceted Rasch Modelling (MFRM), can also be used to identify sources of error (e.g., examiner, domain and station) which may influence the student outcome [67]. These statistical approaches are used *post hoc*, after an assessment, and may offer opportunities to moderate marking. Whether this is possible or not will depend on the wider context. In some countries, information on personal characteristics (e.g., ethnicity, gender) is collected and protected but can be accessed for legitimate purposes which adhere to ethical and governance processes. In other countries, the legal stance is that information on ethnicity and race cannot be collected, thus meaning this type of research is not possible.

##### Guideline 3.

*Sample broadly across assessment types and assessors to minimize bias* **(Strong)**

There is ample evidence that assessor judgements involve a degree of subjectivity particularly in clinical assessments [68–70], and examiner variability is a significant source of variability in OSCE scoring [71–74]. Many (more positivist) studies have raised the possibility that examiner bias may be a contributing factor to minoritized students performing poorly on assessments [75–77]. However, the outcomes of studies looking at examiner bias in medical education are inconclusive. A study in one UK medical school found that student ethnicity did not influence examiners’ scores or feedback [78]. Other studies have suggested that conscious or unconscious assessor biases can impact on the assessment process [79, 80], with one study concluding that “we cannot confidently exclude bias from the examiners in the way that they assessed non-white candidates” [81].

Alternative literature has explored the opposing idea that subjectivity should not and cannot be removed from assessment [82–84]. Such literature acknowledges and accepts examiner variability as not only unavoidable, but also as a potential source of greater defensibility and validity of assessment judgements [84].

This perspective promotes the notion of broad sampling in assessment as a guiding principle in assessment systems. Broad sampling involves the conscious use of multiple assessments utilizing different assessors and on multiple occasions. This approach to assessment is a key cornerstone of programmatic assessment [83, 84], and has been shown in one study to reduce ‘ethnicity-related differences in grades’ [81]. While broad sampling cannot be expected to fully reduce any latent examiner bias, the use of multiple assessment moments may also expose discrepancies stemming from those judgements about students’ attainment [80]. Interestingly, we could not find any literature which directly investigated whether “broad sampling” of assessors should deliberately include “diverse assessors.” However, drawing on the literature more broadly indicates the need to do so [85].

##### Guideline 4.

*Include the concept of ‘cultural validity’ in the design, development and review of assessments* **(Moderate)**

This recommendation derives from wider literature which explores the impact of social background on academic attainment, with particular attention to the representation (or not) of minoritized students in assessment activities, and the way minoritized students view and interpret assessment tasks [86]. This guideline is related to Guideline 5 sharing the position that the nature of the students’ experience of the curriculum is central to their learning and attainment but focusing specifically on the nature of the assessment tasks themselves.

The concepts of culturally valid and responsive assessment [87, 88] challenge assumptions that a test designed for and implemented under standardized conditions is inherently fair or appropriate for all learners [89, 90]. Instead, (written and clinical) assessments should reflect the diversity of the test-taking population, minimizing the risk that students might feel excluded or alienated. This can be done by: considering the ethnic characteristics of people represented in assessments and whether there is any stereotyping of minoritized groups (unintended or otherwise); simulated patient and scenario ethnicity; the positioning of minoritized people in relation to majority members; and the inclusivity of the language used [59].

Minoritized students may have different experiences and different ways of viewing the world, or epistemologies, which impact on how they approach learning and respond to assessments [91–93]. It is not acceptable to assume that the responsibility lies with minoritized students to exclusively “accommodate” their ways of knowing to assumed “normative” assessment practices [89, 94]. Accordingly, assessment review practices need to consider how items may be viewed from the minoritized perspective, both affectively and cognitively. Involving minoritized students and faculty in assessment planning and review may be one approach to address this issue [89] (but please see Guideline 9 for a caveat).

##### Guideline 5.

*Recognize unequal privilege and power in the learning environment as causes of differential attainment and take steps to mitigate their negative impacts* **(Strong)**

There is a strong and consistent body of literature describing discrimination faced by racially-minoritized medical students. This racism occurs at personal and structural levels.

At a personal level racism is experienced in the form of microaggressions [95, 96], stereotype threat [97] and harassment from peers, patients and faculty [98]. At a structural level, racism operates through biased curricula [99, 100], and knowledge production [3] in which Eurocentric knowledge and practices are valued above others.

Given the discrimination that minoritized students face, we must consider personal and institutional racism within the learning environment as causes of differential attainment. Indeed, a review of 28 studies concluded that minoritized students reported less positive learning environments and were more likely to experience racial harassment compared with non-minoritized students. Across these studies academic achievement was worse and academic progress slower for minoritized students [50]. More recent studies indicate that things have not changed notably in the 10 years since Orom and colleagues completed their review [95, 97].

What can we do differently in the future to lead to improvements? There are some studies exploring what might make a difference. Being part of an established social network and building relationships with faculty are correlated with (positive) medical student experiences and outcomes [101]. Having access to both formal and informal social networks are important building blocks for creating social capital [49, 102] and a sense of belonging [103, 104], a lack of which is also associated with poor achievement and discontinuation of studies [105].

##### Guideline 6.

*Medical schools must take responsibility for their role in creating and perpetuating differential attainment* **(Strong)**

Inequality in the broader societal context can impact on attainment within medical education. Minoritized students often carry a burden of historical disadvantage with them when they enter medical school [41]. Universities may further exacerbate this disadvantage through discriminatory systems and practices [48].

One example of this is “deficit thinking”, where there is an implicit or explicit assumption that minoritized students are in some way deficient and institutions hold minoritized students responsible for the inequalities and challenges they experience [2, 106]. As a starting point to addressing differential attainment, medical schools must seek to recognize and revise systemic deficit thinking, as this will allow them to become aware of and take responsibility for factors in their curricula, policies, learning environments and physical environments that contribute to discrimination and differential attainment [4]. There is, however, little practical guidance on how to do so—we hope these guidelines will go some way to helping medical schools reflect on their practices.

##### Guideline 7.

*Recruit and promote under-represented faculty* **(Strong)**

A diverse faculty can contribute to the creation of equitable inclusive structures and practices that will support equitable student outcomes [42]; bring diverse perspectives to curricula and assessments [48]; and provide representative role models.

The importance of learning from role models has long been recognized within medical education [107–109]. Role models are influential in terms of career and specialty choice [110], developing clinical knowledge and skills, as well as understanding the culture of medicine [49, 111]. Students identify more strongly with role models who are similar to them in some way [112, 113] and there is value in having role models from the same ethnic background as students [114, 115]. However, diversity of ethnic backgrounds is often under-represented in faculty, especially in senior positions, giving rise to a paucity of role models for medical students [98, 116–120]. We suspect, but do not know, that the lack of representation and role models within the broader learning environment may impact on the learning and performance of minoritized students [78, 121, 122].

Importantly, research shows that under-represented faculty also experience bias and discrimination [123, 124], and greater difficulty in achieving promotion and advancement [125–128]. They are also more likely to feel isolated and less satisfied with their career, professional development and networking opportunities [123, 127, 129–131]. Medical schools must consider how best to support under-represented faculty via, for example, support from senior leaders [127, 132], peer networking [127, 132], professional skills development [132] and mentoring programmes [112, 133–135]. Campbell et al. [135] point out that faculty development initiatives for under-represented faculty must be complemented by initiatives that address inequalities in opportunities and recognition and foster an inclusive culture and environment [126]. Price et al. [131] advocate that institutions undertake a formal assessment of their ‘diversity climate’ in order to better understand and then inform organizational changes.

#### Don’ts

##### Guideline 8.

*Don’t assume that minoritized students will have the same exposure to and familiarity with particular assessment methods as their non-minoritized peers* **(Tentative)**

This guideline builds on the concept of sampling broadly across assessment types (Guideline 3) and specifically recognizes that educators cannot assume that minoritized students will have had the same exposure to and experience of particular assessment methods as their non-minoritized peers [41]. Differences in prior experience may impact on actual performance, and therefore may contribute to relative underperformance compared with students who are familiar with those methods [80, 120]. Greater use of formative assessments for minoritized students, to both improve familiarity with specific assessment formats and, arguably even more importantly, to alter minoritized students’ sense of agency with particular assessment systems and their own role in the learning process, may be helpful [87, 136].

##### Guideline 9.

*Don’t implement ‘diversity’ practices which place unacknowledged and unrewarded burdens on minoritized faculty and students*** (Moderate**)

Under-represented faculty (i.e., faculty from minoritized groups) are often called upon to work on diversity initiatives, mentor minoritized students and applicants, and serve on equity and diversity committees [132, 137]. Such activities are often not valued in terms of promotion, and time spent on such activities takes faculty away from pursuits which are more beneficial in terms of individual career progression [138]. This often-unacknowledged burden has been described as a ‘minority tax’ [128, 135, 137, 139–141]. Minoritized medical students can also experience minority tax, feeling pressure to take part in activities such as mentoring or outreach activities for minoritized students [117]. It is challenging to balance engagement with minority taxation but, as per Guideline 6, change at a systems level is required so these activities are appropriately recognized and rewarded.

Other faculty can also address some of this unbalanced workload by challenging discrimination, supporting under-represented faculty and actively participating in initiatives to address lack of diversity amongst faculty and students [140, 141]. To contribute effectively in this way, faculty development could usefully include: understanding different forms of discrimination such as implicit bias and microaggressions [135]; learning how to challenge racism and examining everyday practices that reinforce existing power structures [103]; and mentoring minoritized students [137], engaging in critical reflection [142] and recognizing when to refer students to others [112].

##### Guideline 10.

*Don’t attribute to the individual what is systematic* **(Strong)**

As per Guideline 7, increasingly, differential attainment is recognized as a systemic issue and not an individual one [2, 106]. That prolonged disadvantage is an issue is unarguable. Yet historically, efforts at redress have too often focused almost exclusively on interventions that target the individual learner. For example, many schools have responded to this academic achievement divide between minoritized and non-minoritized students by establishing premedical school “enrichment”, gateway or pipeline programmes, to bridge the gap for minoritized students [143]. (Consideration of the assumptions of such programmes and how they may perpetuate deficit models is beyond the remit of this paper). However, as we hope is obvious so far, the past few decades have moved this conversation on to recognize that minoritization within medicine is itself an independent predictor of exam success. Therefore, systematic and structural changes need to take place, rather than merely focusing on individual student success. The terms differential awarding instead of differential attainment, and awarding gap rather than attainment gap have been proposed recently as a way to shift the focus more toward institutional responsibility [21, 53].

#### Don’t knows

##### Guideline 11.

*Are supportive interventions for specific groups effective in respect of assessment outcomes?*

Some educators and researchers argue for targeted interventions for specific groups to address differential attainment [79]. This is an approach which is relatively underutilized in medical education and training (see [144] for an exception) but is reported more extensively in the wider education literature. For example, studies have examined the efficacy of self-affirmation interventions in relation to assessment performance in ethnic minority groups [145, 146]. Another approach reported as effective in terms of addressing differential attainment is that of enhancing goal-directed conceptualization and action [147]. This approach aims to support minoritized students in challenging learning environments to enhance their motivation and self-regulation [148]. Again, however, while promising and encouragingly well-grounded in theoretical principles, neither self-affirmation nor goal-directed approaches/interventions have been tested empirically in minoritized medical students.

More generally, the onus is on medical schools to scrutinize routine assessment and other data to develop targeted strategies in areas of particular concern [149]. Extrapolating from Cleland et al. [150], it is then crucial to evaluate the impact of additional support for specific groups of students to identify what works, from whom and why in terms of assessment support.

##### Guideline 12.

*To what extent do professionalism norms and assessments of professionalism discriminate against minoritized students?*

As described earlier, there is growing evidence that cultural norms and values are embedded in assessment design and practice. If these reflect only the norms of the privileged group, this can result in biased assessments that lack cultural validity and may contribute to differential attainment. In particular, the assessment of professionalism in examinations is likely to reflect culturally embedded values about the practice of medicine [121]. For example, medical educators may have a white Eurocentric view of what clinical communication looks like or how empathy is expressed [116].

Although ethnicity is acknowledged to be a part of one’s personal identity [151], the understanding of the influence of race and ethnicity on the process of professional identity formation (PIF) and its impact on the assessment of professionalism is largely absent from published literature [141, 152–154]. The wider literature suggests areas for further research. For example, socialization is critical in PIF [155]. Given minoritized students have less extensive peer and faculty networks (see Guidelines 6 and 8), it would be worth exploring if and how these may impact on their PIF and progression from student to physician. In trying to make sense of their race or ethnicity and becoming a member of an apparently “white” profession, minoritized students may struggle to see how they can conform to this stereotyped image of a doctor [156, 157] and may conceal or repress aspects of their racial or ethnic identity [138, 158] and/or experience tensions in trying to reconcile these and a new professional identity [159]. Conversely, if dominant professional norms are rejected in this process of PIF, this could have an impact on attainment and progression through medical education and training. Direct evidence is lacking, but in many countries doctors from minority groups tend to receive disproportionately more sanctions or warnings than those identifying as from the dominant group [160, 161]. Retrospective and prospective studies which examine patterns of performance and explore underlying reasons for differential attainment are needed.

Research is also needed to understand how aspects of student identity relate to experiences of minoritization within medical education [162], and how this in turn relates to differential attainment.

##### Guideline 13.

*How can formal curricula influence differential attainment?*

Research from higher education posits that curricula are important to redressing differential attainment [48]. Inclusive pedagogies and curricula are theorized to combat the centrality of whiteness and thus experiences of minoritization within higher education [3, 4, 163]. Two particular approaches are being implemented within medical education: inclusive curricula and removing racism from curricula.

Inclusive curriculum strategies aim to make curricula accessible and acceptable for all students [4]. These include “decolonizing” curricula by bringing different voices and knowledge sources into courses (epistemic pluralism) [4]. Some medical schools are actively undertaking work to ensure previously unheard voices (voices that legitimize issues of gender, race and class) within their curricula and to address issues such as ethnic biases in cases and teaching materials [34, 99, 164–167]. Future research could examine the processes of strategy implementation [168] and strategy impact on the learning environment, student experience and differential attainment.

Throughout their curricula, medical schools teach directly and indirectly about race, ethnicity and health. Race-based medicine positions race as a biological variable that influences physiological functioning and thus becomes a basis for differential clinical care [169]. Biological conceptualizations of race, which are deeply embedded in medical education [99], contribute to institutional racism impacting on student learning, and ultimately racial health inequalities [99, 170]. “Anti-racist” curricula shift teaching about race as a biological variable to that of a social construct, thus prompting explanations about health inequalities that focus on equity and discrimination rather than spurious genetic explanations [99, 164, 171–173]. There is the potential for anti-racist curricula to re-frame conceptions and misconceptions of race and racism, that impact on values, cultures and norms [163].

However, empirical evidence as to the effectiveness of curricular strategies to redress differential attainment is currently lacking.

## Conclusion

Differential attainment is a complex, systemic problem reflective of unequal norms and practices within broader society and evident throughout assessment practices, the learning environment and student experiences at medical school linked to systems/institutional factors (Table 2). This paper summarizes what we currently know from the published literature and our own knowledge and experiences of differential attainment. These guidelines reflect the core values of education, highlighting the importance of fair and transparent educational policies, practices and structures, as well as our societal responsibility to learners and patients.

It is clear that the most convincing evidence is that around assessment processes themselves, arguably because this is the most tangible area to focus on in respect of addressing differential attainment. However, there are issues with some of this (mostly quantitative) research in terms of how learners are categorized, limiting granularity and knowledge. The evidence on minoritized students (and faculty) facing discrimination in their environment and different experiences is emerging, but more studies are needed and there is a pressing need for research on how to address systemic issues. We also suggest that further research should focus on those areas where the evidence for the recommendations is not strong or has not been researched over the longer term, where intuitively recommendations seem sensible, but have not been fully substantiated and explored, or those areas which have been identified as “Don’t Knows”.

The good news is that, as medical educators, we have increasing awareness of and expertise in practices that can address differential attainment and minoritization. Differential attainment is not just an issue for those involved in assessment: it must be tackled at an institutional level. Ultimately, decisions about differential attainment reflect institutional values and, therefore, clarifying those values is critical. Indeed, our intention is that this summary of the current state of differential attainment research will enable individuals, institutions and the medical profession to make more informed choices about how to support all learners. There is no simple way of doing so but we suggest that these guidelines can be the basis of critically examining (both quantitatively and reflexively) whether certain practices and structures privilege certain groups. The trajectory of reflection which will result from this exercise can be used to question “the given”, and by doing so “interrupt” complicity and ongoing reproduction of differential attainment and minoritization.

This is a growing area of research—there are far fewer studies on differential attainment in medical education than there are studies in, for example, remediation [39] or feedback [40] and thus and key questions remain unanswered. It is also important to note that we identified very few studies from low- and middle-income countries [110, 115] and all empirical studies on differential attainment originated from the USA, the UK, Canada, Europe and Australia. This may have been due at least to some extent to our search being limited to English-language publications but there does seem to be a global gap: medical schools in lower-income countries are either not publishing on differential attainment in medical education, not publishing in English and/or not publishing in journals listed in mainstream databases. This absence in the literature is important in terms of “how we construct the field of health professions education research: what we include or exclude, what we count or not, what we believe to be true or false, what we do or do not read, who speaks and who is silenced” (Paton et al., 2021, p. 6. [174]). The global medical education community knows little about differential attainment practices and ideals in low- and middle-income countries, and there is no opportunity to learn from different ways of thinking and doing.

However, we are optimistic that wider societal drivers, such as Black Lives Matter, are increasing awareness within medical education globally, and this increased awareness will lead to action and evaluation so that an update of this review in 10 years’ time will be able to synthesize many more studies. With change will come the need to evaluate change—not just in terms of outcomes for particular minoritized groups, but also to ensure we understand the processes of change and can monitor for any unintended consequences for certain individuals or groups. We make a plea for research approaches that capture the complexity and nuance of intersecting identities and how these may influence the experience of education and assessment.
